# SYRMEP Tomo Project: a graphical user interface for customizing CT reconstruction workflows

**DOI:** 10.1186/s40679-016-0036-8

**Published:** 2017-01-19

**Authors:** Francesco Brun, Lorenzo Massimi, Michela Fratini, Diego Dreossi, Fulvio Billé, Agostino Accardo, Roberto Pugliese, Alessia Cedola

**Affiliations:** 1 National Research Council-Institute of Nanotechnology (CNR-Nanotec), c/o University La Sapienza, P.le Aldo Moro, 5, 00185 Rome, Italy; 2 Department of Engineering and Architecture, University of Trieste, Via A. Valerio, 6/1, 34127 Trieste, Italy; 3 Elettra-Sincrotrone Trieste S.C.p.A., S.S. 14 km 163.5 in Area Science Park, 34149 Basovizza, Trieste Italy; 4 Fondazione Santa Lucia, Via Ardeatina, 306, 00179 Roma, Italy

**Keywords:** Phase-contrast computed tomography, Tomographic reconstruction, Image processing, Phase retrieval, Artifacts compensation

## Abstract

When considering the acquisition of experimental synchrotron radiation (SR) X-ray CT data, the reconstruction workflow cannot be limited to the essential computational steps of flat fielding and filtered back projection (FBP). More refined image processing is often required, usually to compensate artifacts and enhance the quality of the reconstructed images. In principle, it would be desirable to optimize the reconstruction workflow at the facility during the experiment (beamtime). However, several practical factors affect the image reconstruction part of the experiment and users are likely to conclude the beamtime with sub-optimal reconstructed images. Through an example of application, this article presents *SYRMEP Tomo Project* (STP), an open-source software tool conceived to let users design custom CT reconstruction workflows. STP has been designed for post-beamtime (off-line use) and for a new reconstruction of past archived data at user’s home institution where simple computing resources are available. Releases of the software can be downloaded at the Elettra Scientific Computing group GitHub repository https://github.com/ElettraSciComp/STP-Gui.

## Background

Synchrotron radiation (SR) X-ray Computed micro-Tomography (${\upmu }$-CT) in its simplest form deals with the inversion of the Radon transform of acquired parallel beam projection data. In this case, the application of an implementation of the filtered back projection (FBP) algorithm after the so-called *flat fielding* of the projection data is often proposed as a solution for the tomographic problem. This approach is formally correct and, in general, easy to apply since most of the computing software tools used by scientists (e.g., MATLAB^®^, Mathematica^®^, IDL^®^) offer an implementation of the inverse Radon transform. However, this approach is based on the traditional way to extract contrast in X-ray radiography, i.e., the consideration of the different attenuation properties of the elements composing the imaged object. Such properties are related to $\beta$, the complex part of the index of refraction $n = 1- \delta +i\beta$. Thanks to the coherence of SR, quantities other than absorption, like the electron density, related to $\delta$ might be exploited leading to phase-contrast imaging [1].

Among the phase-contrast modalities, single-distance propagation-based imaging (PBI) is one of the most exploited in SR $\upmu$-CT experiments as it is based only on the relative distance between the rotating sample and the detector. According to this modality, neither hardware elements (e.g., crystals or gratings) nor multiple acquisitions are required to derive information about the phase shifts induced by the imaged object. In this case, rather than performing the conventional reconstruction of the attenuation coefficient (that would result in the so-called *edge enhanced* image), a phase retrieval algorithm is typically applied to the (flat-corrected) projection data in order to reconstruct the decrement from unity of the refractive index.

The main motivation behind the efforts of reconstructing the $\delta$ is to differentiate between two materials of similar electron density or with negligible X-ray absorption (i.e., phase objects) so as to ease the image segmentation step and the subsequent analyses. An example is the differentiation of soft tissue in biomedical applications which is typically better imaged when considering the contribution of the phase information [2]. More recently, thanks to significant improvements in the acquisition speed, fast time-resolved tomography is also performed at SR sources to image “dynamic” samples. In such cases, the short exposure time applied results in a limited photon flux. Accordingly, the consideration of $\delta$ instead of $\beta$ becomes particularly interesting to enhance image contrast by taking advantage of the (stronger) refractive effect rather than attenuation. Phase retrieval can be viewed as a tool for the relaxation of the photon flux requirements in order to increase the time resolution of ultra-fast SR $\upmu$-CT experiments still preserving a good contrast [3]. Similarly, a limited photon flux is considered when the radiation dose is a concern, such as e.g., in sight of in vivo application of SR CT [4]. Therefore, it is not incorrect to state that the essential reconstruction pipeline required by today PBI SR $\upmu$-CT experiments is a three-step process composed by flat fielding, phase retrieval, and the actual reconstruction.

While several algorithms have been proposed in the last decades for (approximating) the retrieval of phase information from only one measurement [5], the currently most used method has been proposed by Paganin et al. [6]. This method established itself basically because it is fast (being non-iterative) and also stable with respect to noise. Moreover, its main assumption is the homogeneous material composition of the imaged sample. The Paganin’s algorithm assumes that the phase and the amplitude of the incident wave are related to a known ratio $\delta / \beta$. This simplicity is an additional advantage of the Paganin’s algorithm.

Although the tomographic reconstruction of the acquired data can be performed at a later stage at user’s home institution, in a PBI SR $\upmu$-CT experiment, a highly desirable goal is to perform a preliminary reconstruction and visualization of the data during the beamtime. This enables rapid feedback on data quality and experimental conditions as well as sample preparation and positioning onto the rotating stage (a tricky task when scanning pure phase objects at very high resolution). To this aim, a $\upmu$-CT beamline typically offers software to apply the already mentioned “standard” fast reconstruction pipeline composed by conventional flat fielding, Paganin’s phase retrieval and FBP. In this case, in addition to the practical issue of the assessment of the center of rotation [7], the only parameter to tune is the $\delta / \beta$ of the phase retrieval step but it requires only a few attempts with visual supervision. By considering this workflow as “the” solution to the tomographic problem, users typically invest their time and efforts during the beamtime in the optimization of the acquisition parameters (e.g., beam energy, sample-to-detector distance, and number of acquired projections) and in the maximization of the number of scanned samples. If users are satisfied with the reconstructed data produced with this approach in terms of image quality, thanks also to high-performance computing (HPC) hardware typically available at the facility, they are likely to conclude the beamtime with the reconstructed data for all the scanned sample. Post-beamtime refinements and optimizations can be performed only if access to a remote computing infrastructure is offered by the facility. An interesting example is the Australian MASSIVE initiative [8]; however, most of the European facilities do not offer similar remote tools for data processing and visualization.

On the other hand, some users may want to push the frontiers of their research looking for the best image quality achievable in order to ensure an accurate quantitative analysis of the reconstructed volume. They may want to invest time and efforts in the optimization of the reconstruction process by testing non-conventional flat fielding solutions, different phase retrieval algorithms as well as enhanced reconstruction approaches, such as algebraic techniques. For instance, the most recurrent additional pre-processing step deals with the compensation of ring artifacts that hamper the interpretation and segmentation of the reconstructed data [9]. Efforts in the optimization of the ring artifacts compensation step require several attempts. Also intensive computational time is required by some of these approaches, thus leading to slow reconstruction workflows (slower than the time required by data collection). Moreover, in a large scientific collaboration, it is not uncommon that the researchers involved in the data acquisition at the facility are different from those involved in the interpretation and analysis of the reconstructed images. These researchers, more expert in the image evaluation, might not possess programming skills and, therefore, their contribution can be discouraged if they are required to modify, e.g., MATLAB^®^, IDL^®^ or Python scripts. An interesting additional point is that, as research in digital image processing goes on, users might be interested in applying new algorithms to past archived datasets. This is particularly true for experiments where rare specimens are scanned [10] or investigations where destructive complementary analyses (e.g., histology) are considered after the $\upmu$-CT acquisition. In this case, it might be hard (or even impossible) to perform a new data collection. In this scenario, the availability of user-friendly software tools for a post-beamtime optimization of the reconstruction workflow at user’s home institution might increase the image quality and improve the subsequent analyses.

This article presents *SYRMEP Tomo Project* (STP): an open-source software tool with a graphical user interface (GUI) conceived to let users design custom CT reconstruction workflows. In the next section, the software architecture and the design principles are presented. Then, an application is reported where the “standard” reconstruction workflow (conventional flat fielding, Paganin’s phase retrieval and FBP) is compared in terms of image quality against a custom workflow applied by taking advantage of some of the features of STP. Releases of the STP software can be downloaded for free at the Elettra Scientific Computing group GitHub repository https://github.com/ElettraSciComp/STP-Gui.

## Software architecture

**Fig. 1:**
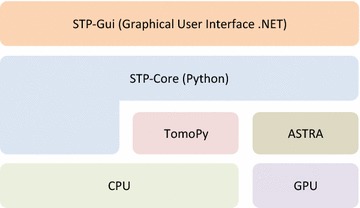
*SYRMEP Tomo Project* software architecture: the graphical user interface (GUI) is separated from the core functions. *STP-Core* takes advantage of *TomoPy* and the ASTRA toolbox

*SYRMEP Tomo Project* (STP) has been designed with a strong distinction between the GUI and the core functions. Figure 1 shows the two main blocks composing the software, namely *STP-Gui* and *STP-Core*. The GUI is built on top of an internal project [11], recently re-named *STP-Core* developed in Python language. The *STP-Core* functions are used at the SYRMEP beamline (Elettra-Sincrotrone Trieste S.C.p.A) [12] for a fast online (i.e., during the beamtime) reconstruction. Among the motivations behind the choice of the Python programming language, there is its cross-platform nature. With limited efforts, all the *STP-Core* functions can be also, in principle, executed in a Unix-based HPC computer cluster. The Python programming language was chosen also to favor the interaction with the *TomoPy* initiative [13] and the ASTRA toolbox [14], for which Python wrappers are available. The ASTRA toolbox provides highly efficient tomographic reconstruction methods implemented for graphic processing units (GPUs). ASTRA is only focused on reconstruction, and it does not include pre- or post-processing methods. Furthermore, no routines to read data from disk are provided by the toolbox. On the other hand, *TomoPy* includes several pre-processing and post-processing algorithms commonly used for synchrotron data as well as different reconstruction algorithms. The algorithms implemented in *TomoPy* are all CPU-based, and this can make them prohibitively slow when considering the size of datasets commonly produced by a PBI SR $\mu$-CT. The two toolboxes have been also recently integrated [15] into a common framework but still a GUI is missing (users are required to customize a Python script to invoke the features).

**Fig. 2:**
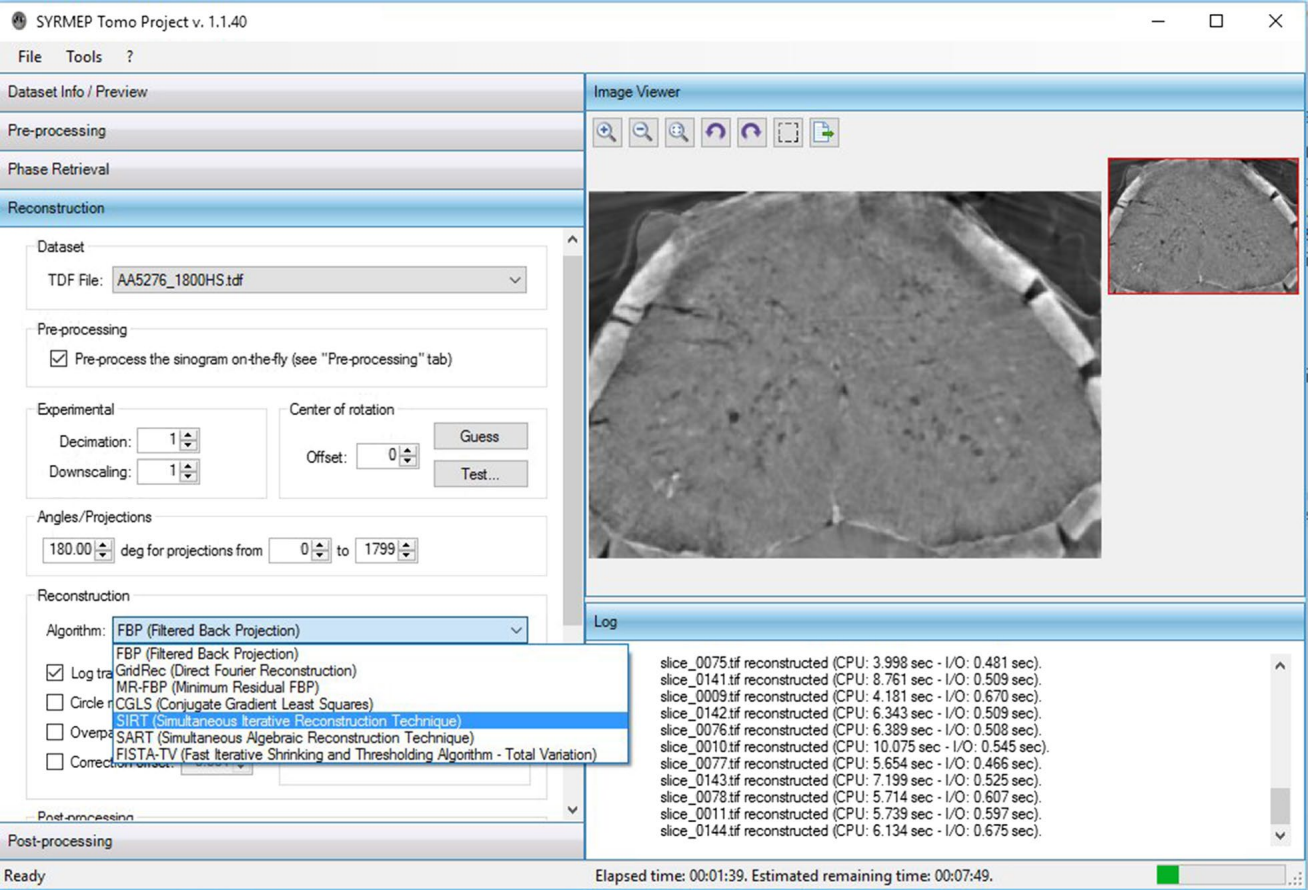
Snapshot of the STP main window. The image preview reports a zoom in on a reconstructed slice of a healthy adult male C57 Black mouse (20–22 g, body weight) spinal cord at the lumbar level. The acquisition (energy = 30 keV, sample-to-detector distance = 2200 mm, nominal image pixel size = 3.05 $\upmu$m) was performed at the ID17 beamline of the ESRF (Grenoble, France)

The *STP-Gui* module is developed in C# .NET language. The only motivation behind this choice is related to the programming skills of the main developer involved in the project. *STP-Gui* has been specifically designed to be used with Microsoft Windows^®^ operating systems. The user interface is composed of a sidebar where the common steps of a CT reconstruction pipeline are reported (see Fig. 2). For each of these steps, a job can be defined, which means that intermediate output can be generated, in order to let users exploit external tools or collaborations for maximum customization of all the steps of the reconstruction workflow. The remaining part of the main window is composed of an image viewer (where the basic functionalities of zoom in/out as well as brightness/contrast settings are available) and an output section where the running job reports information to the user. By modifying the available options in the graphical user interface, users customize the job to be submitted to the local machine. While the current job is running, users can “tune” the next task to be submitted by testing different options and previewing the output.

*SYRMEP Tomo Project* aims at offering state-of-the-art digital image processing solutions for the reconstruction of phase-contrast PBI SR $\upmu$-CT datasets. To simplify the extension of the software with new tools, some portions of the GUI can be easily customized by Python programmers. For instance, new ring artifacts removal filters can be added to *STP-Core*. Python programmers are required to follow basic conventions when writing their code, and they are required to extend a simple XML file in order to have the new feature visible from the user interface. Without recompiling the *STP-Gui* module, custom filters become available as soon as the modified feature is re-invoked.

## Application

Since PBI SR $\upmu$-CT allows to reconstruct the internal structure of a sample at a range of scales spanning from hundreds to a few microns, it has a strong impact in a large number of pre-clinical investigations, such as in the case of the neuro-degenerative pathologies. In this framework, it is very important to reconstruct high-quality images in order to derive accurate quantitative and qualitative information. In the application presented herein, the “standard” reconstruction workflow (conventional flat fielding, Paganin’s phase retrieval and FBP) is compared in terms of image quality against a custom workflow. The considered sample is an excised mouse brain embedded in agar and imaged at the ID17 beamline of the European Synchrotron Radiation Facility (ESRF) with the following experimental condition: energy = 40 keV, sample-to-detector distance = 2200 mm, nominal image pixel size = 3.05 micron, 4000 projections collected according to the so-called half acquisition mode (i.e., an off-center rotation over 360$^\circ$ in order to almost double the width of the field-of-view) [16], and exposure time = 0.3 s per projection.

**Fig. 3:**
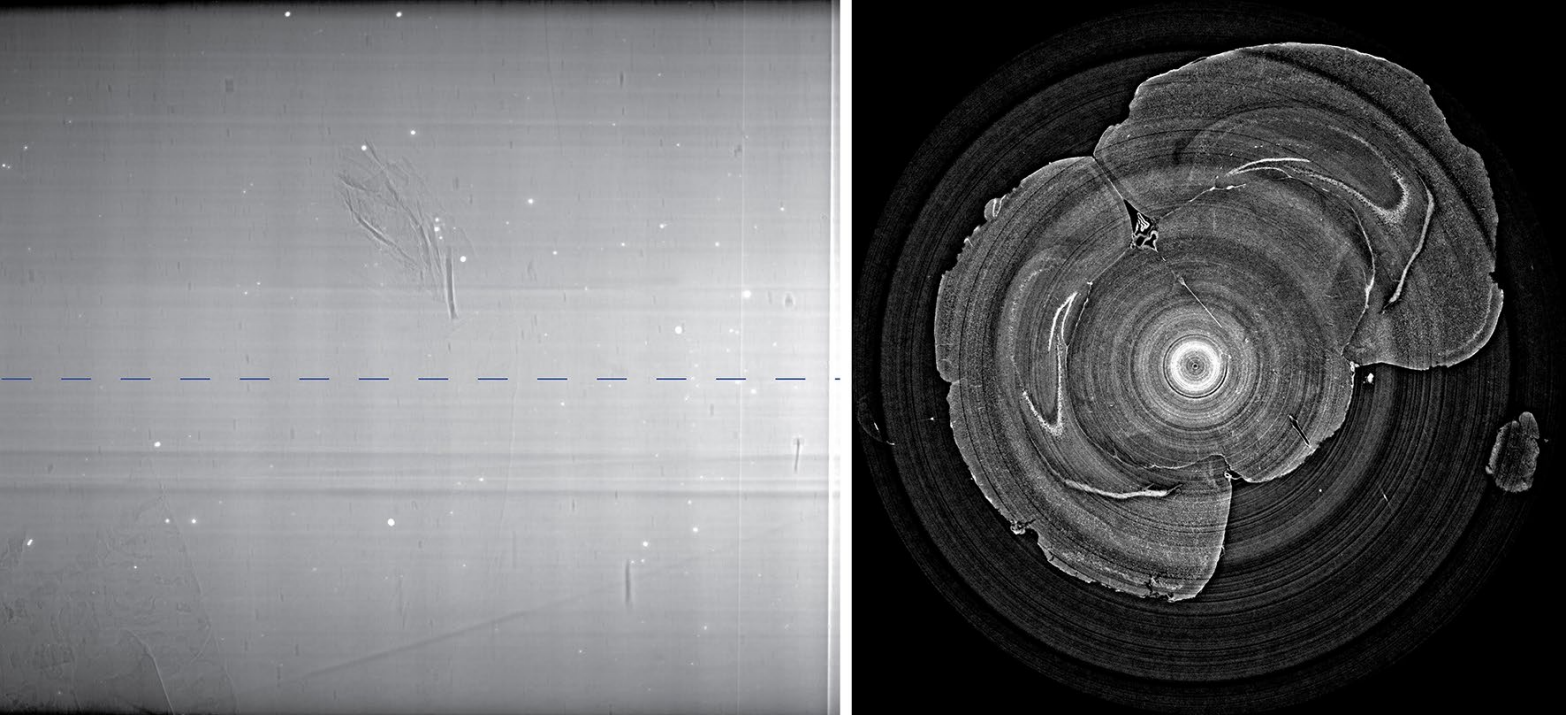
On the *left*: sample projection as acquired by the scintillator detector system. On the *right*: reconstructed slice (corresponding to the *line* denoted in the projection) with the fast conventional protocol (flat fielding, Paganin’s phase retrieval, and FBP) as produced during the beamtime by the PyHST software [17] and the help of beamline personnel

Figure 3 presents an acquired projection and an axial slice reconstructed with the conventional protocol during the beamtime. The slice was reconstructed with the PyHST software [17] and the help of beamline personnel. Very few details can be noticed in the acquired projection, thus confirming that the scanned sample is almost a pure phase object. Some spurious absorbing elements (most likely small skull bone fragments due to imperfections in the surgical excision of the brain) can be noticed in the reported projection. The reconstructed slice (with brightness/contrast set to emphasize the interesting features of the considered sample) presents severe ring artifacts.

**Fig. 4:**
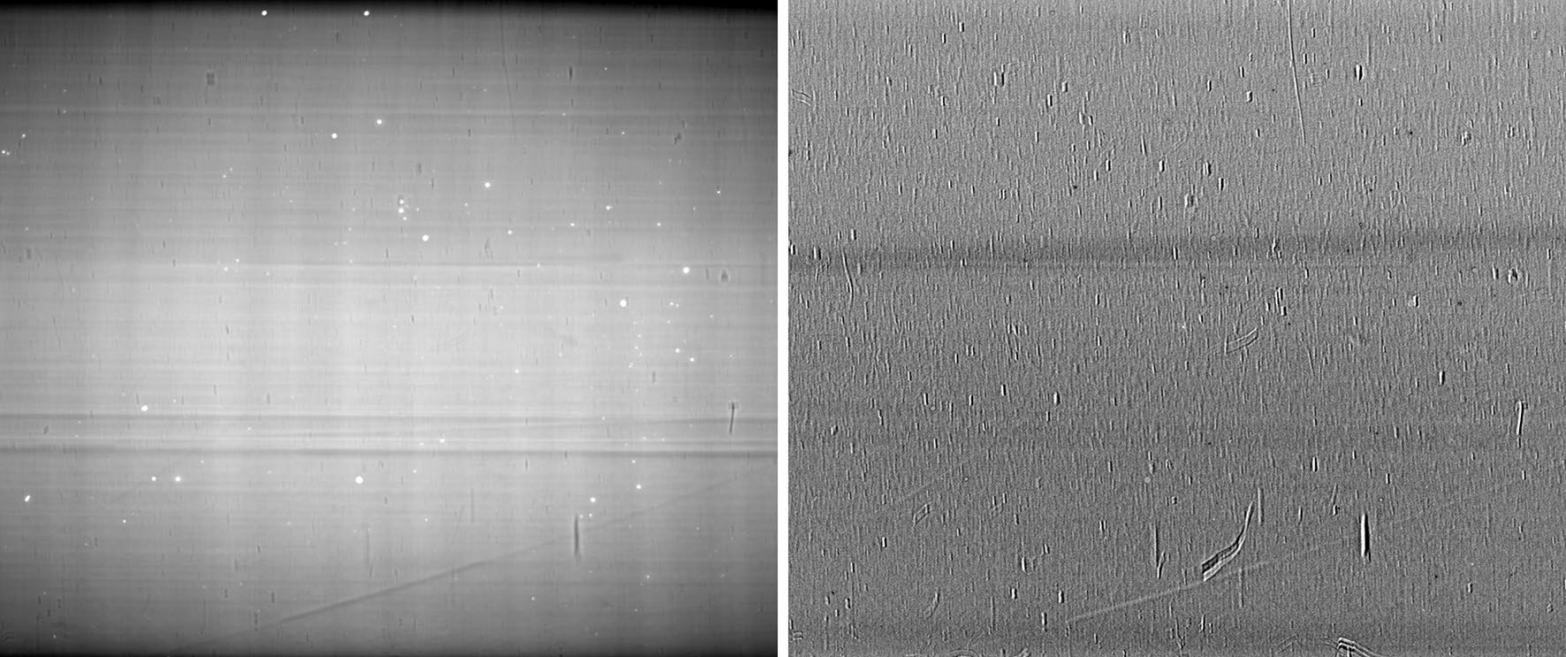
On the *left*: (average) flat field image collected before the acquisition of all the projections. On the *right*: pixel-by-pixel difference between the (average) flat field image taken before the acquisition of all the projections and the (average) flat field image collected after the full rotation of the sample (not reported). The slight variations of the flat field images due to the instabilities of the X-ray source and other beamline components (e.g., the monochromator) can be noticed

In order to understand some of the reasons behind the challenges involved in the reconstruction of the considered dataset, let us consider Fig. 4. It reports an acquired flat field image as well as the pixel-by-pixel difference between the (average) flat field image taken before the acquisition of all the projections and the (average) flat field image collected after the full rotation of the sample. While, in principle, intermediate flat fields can be collected, when dealing with very high-resolution imaging, it is preferable to avoid the repositioning of the rotating stage if a complete set of projections is not acquired. By observing the reported flat field image, bright spots (induced by the detector scintillator system) are clearly visible. The slight deviations from zero visible in the difference image suggest also an additional source of artifacts, i.e., the instabilities of the X-ray source and other beamline components (e.g., the monochromator). These variations are emphasized by the long scanning time required for the considered sample. A refined reconstruction workflow is therefore required to mitigate the artifacts that strongly degrade the quality of the reconstructed image and hamper further analyses.

The first step of the custom protocol presented herein is the recently proposed *dynamic flat fielding* [18]. This method is based on the concept of eigen flat fields computed through principal component analysis of the set of collected flat images. In the implementation available in STP, a linear combination of the most important eigen flat fields is used to individually correct each X-ray sinogram. While the method performs better with a high number (i.e., hundreds) of collected images, benefits have been observed also with a few (i.e., dozens) flat field images. A mandatory additional image processing step is the sinogram stitching required by the so-called half acquisition mode. This action results in a dataset being no more a set of images collected over 360$^\circ$, having size equal to the detector field-of-view, but a set of projections collected over 180$^\circ$ whose width is almost two times the width of the original images.

**Fig. 5:**
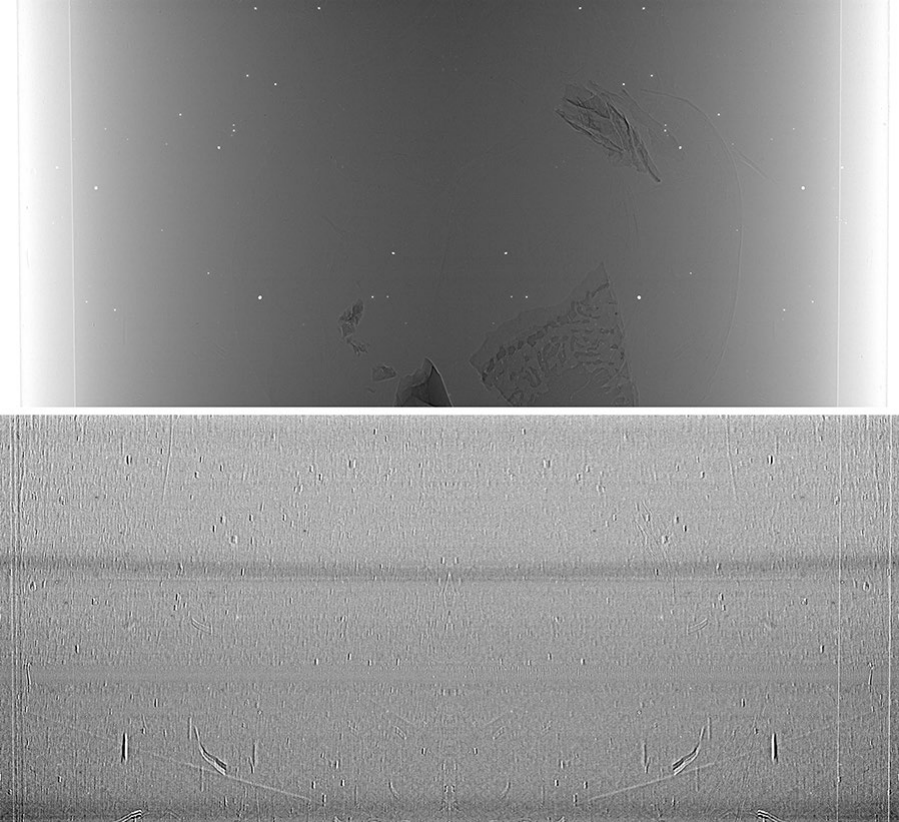
On *top*: sample projection (after sinogram stitching) corrected with dynamic flat fielding. On *bottom*: pixel-by-pixel difference between the same image corrected with conventional flat fielding (not reported)

In order to correctly perform the sinogram stitching, the center of rotation (which is close to either left or right side of the detector field-of-view) has to be assessed with either automatic suggestions (see e.g., [7]) or visual supervision. At the end of this step, the modified dataset is, in principle, ready for conventional (absorption) tomographic reconstruction. Figure 5 presents a corrected projection (after sinogram stitching) with dynamic flat fielding. The pixel-by-pixel difference between this image and an image processed with conventional flat fielding is also reported in order to appreciate the slight improvement produced by the considered method. It can be noticed that bright spots are still present, and this will lead to severe ring artifacts in the reconstructed images.

Ring artifact compensation is usually performed by de-striping the sinogram image prior to the actual reconstruction. Interesting solutions have been also proposed where the stripe removal is applied to a reconstructed slice by including a Cartesian-polar transformation [19]. When considering PBI SR $\upmu$-CT data, common sinogram de-striping approaches can be applied either before or after (or also, two times before and after) the phase retrieval step. In the reconstruction protocol presented in this article, all the sinogram de-striping filters offered by STP [20–23] have been tested. The modified Raven’s approach [20] implemented in STP has been used here to mitigate ring artifacts. The parameters of the filter have been tuned by visual assessment.

Single-distance phase retrieval has been applied prior to the actual reconstruction. In addition to the Paganin’s [6] approach based on the transport of intensity equation (TIE) for phase retrieval, STP offers also an implementation of the *projected CTF* (also called *quasiparticle*) method [24] based on a modified contrast transfer function (CTF) model. This approach is supposed to behave better when dealing with pure phase objects. More precisely, while the TIE approach still produces acceptable results, its stability comes with a recognized associated loss of spatial resolution due to its essential behavior as a low-pass filter [25]. Sharpening techniques such as a restoration algorithm implemented in *ANKAPhase* [26] as well as more refined approaches (see e.g., [25]) have been proposed to this aim. Similarly, for moderately large phase variations, the projected CTF could better preserve the spatial resolution of the reconstructed images [27]. This benefit, however, requires an adequate selection of the sample-to-detector distance when performing the data acquisition (which was not considered for the application presented in this article) and more user efforts when looking for the optimal tuning parameters of the projected CTF method.

**Fig. 6:**
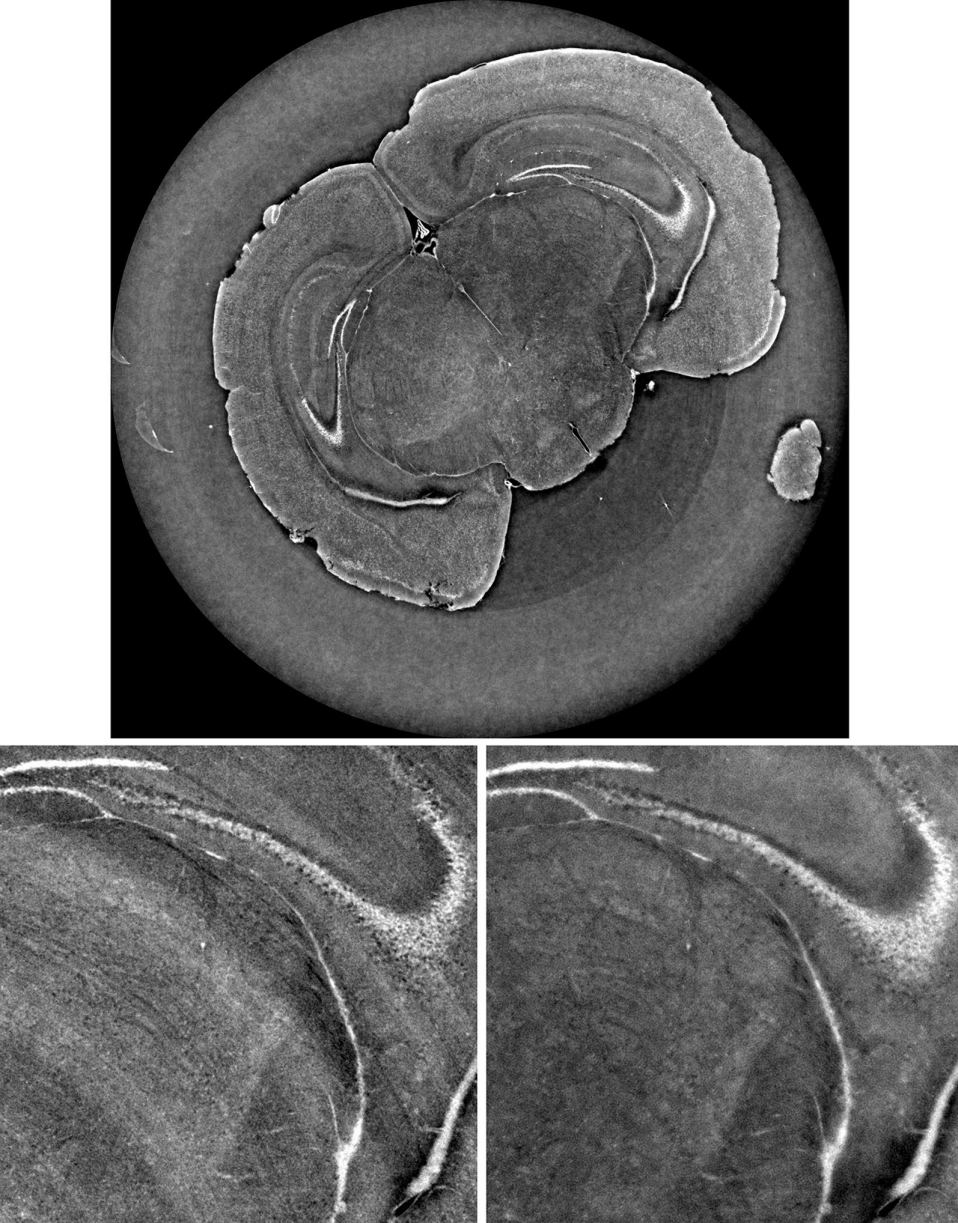
Slice reconstructed with the proposed custom protocol (dynamic flat fielding, ring removal, projected CTF phase retrieval, and MR-FBP reconstruction). Closeup on a ROI extracted from the slice presented in Fig. 3 (conventional protocol: conventional flat fielding, Paganin’s phase retrieval, FBP) [on the *left*] and the same ROI extracted from the slice reported in panel a [on the *right*]

The final step of the proposed reconstruction workflow is the application of a tomographic reconstruction algorithm. FBP is the most widely adopted algorithm in CT reconstruction workflows, and it is generally recognized as the fastest way to produce adequate results when a sufficient number of projections are acquired. However, algebraic methods might be interesting when performing, for instance, experiments in which the radiation dose is a concern, and therefore, a limited number of projections are acquired. In this case, a better contrast-to-noise ratio compared to FBP can be observed [28]. SYRMEP Tomo Project offers users the algorithms implemented in the ASTRA Toolbox [14] in addition to publicly available reconstruction libraries [29, 30]. Figure 6 reports a slice reconstructed with MR-FBP [29] after the above-mentioned pre-processing steps. It can be noticed that ring artifacts have been compensated and spatial resolution is visibly preserved. Although a formal and quantitative comparison with the “standard” fast reconstructed protocol is not taken into account in this article, a global improvement in terms of image quality is obtained for the image reconstructed with a custom suitably tuned digital image processing pipeline. Of course, this improvement required more user efforts and more computational time.

## Future plans

In many cases, a number of similar samples are scanned so it is useful to use a GUI to optimize the reconstruction workflow and then apply the same parameters to a series of datasets. Considering this common scenario, the current version of the software is designed to retain the last adopted parameters. However, it would be desirable to support users with a batch execution, i.e., the preparation of a parameter file describing the reconstruction protocol combined with an automatic application of this protocol to a sequence of datasets without further user support. This feature will be included in future versions of the software.

The link between the GUI and the core features is performed by executing local processes and by monitoring the output of these processes. In principle, the software layer between the GUI and the core features can be replaced with a new layer responsible for redirecting the reconstruction processes to a HPC (assuming that the data to reconstruct is available also on a shared storage resource). This would allow to use the same GUI also for a fast online reconstruction. However, online reconstruction is usually performed with the support of beamline personnel, and it is supposed that expert personnel can operate with command-line tools without a refined graphical interface. At the current stage, the software is designed for execution by non-expert personnel with common hardware at user’s home institution where HPC computing resources are not available. However, future evolutions might consider a transparent integration with Unix HPC to perform a fast reconstruction at the facility.

## Conclusion

Among the phase-contrast modalities, single-distance PBI is one of the most exploited in SR $\upmu$-CT experiments because no additional hardware devices are needed. Multiple data acquisitions are not required; thus, radiation dose and acquisition time are, in general, limited. Therefore the number of scanned samples per beamtime increases, leading to experiments with better statistics or, simply, more experiments. However, in several practical applications, the images produced with the conventional reconstruction workflow for PBI SR $\upmu$-CT might not present an adequate quality for further analyses. These applications can greatly benefit from a flexible reconstruction workflow where refined image processing solutions such as innovative flat fielding approaches, ring artifacts removal filters, enhanced phase retrieval algorithms as well as algebraic iterative reconstruction can be tested. By means of an example of application, this article presented *SYRMEP Tomo Project* (STP): an open-source software tool with a GUI conceived to let users design custom reconstruction workflows without the need of computer programming skills.

## References

[1] Bravin A, Coan P, Coan P (2013). X-ray phase-contrast imaging: from pre-clinical applications towards clinics. Phys. Med. Biol..

[2] Fratini, M., Bukreeva, I., Campi, G., Brun, F., Tromba, G., Modregger, P., Bucci, D., Battaglia, G., Spanó, R., Mastrogiacomo, M., Requardt, H., Giove, F., Bravin, A., Cedola, A.: Simultaneous submicrometric 3D imaging of the micro-vascular network and the neuronal system in a mouse spinal cord. Scientific Reports **5** (2014) 10.1038/srep08514 PMC4649670 25686728

[3] Mokso, R., Marone, F., Irvine, S., Nyvlt, M., Schwyn, D., Mader, K., Taylor, G.K., Krapp, H.G., Skeren, M., Stampanoni, M.: Advantages of phase retrieval for fast X-ray tomographic microscopy. J. Phys. D: Appl. Phys. **46**(49) (2013)

[4] Longo R, Arfelli F, Bellazzini R, Bottigli U, Brez A, Brun F, Brunetti A, Delogu P, Di Lillo F, Dreossi D, Fanti V, Fedon C, Golosio B, Lanconelli N, Mettivier G, Minuti M, Oliva P, Pinchera M, Rigon L, Russo P, Sarno A, Spandre G, Tromba G, Zanconati F (2016). Towards breast tomography with synchrotron radiation at Elettra: first images. Phys. Med. Biol..

[5] Burvall A, Lundström U, Takman PAC, Larsson DH, Hertz HM (2011). Phase retrieval in X-ray phase-contrast imaging suitable for tomography. Opt. Express.

[6] Paganin D, Mayo SC, Gureyev TE, Miller PR, Wilkins SW (2002). Simultaneous phase and amplitude extraction from a single defocused image of a homogeneous object. J. Microsc..

[7] Vo NT, Drakopoulos M, Atwood RC, Reinhard C (2014). Reliable method for calculating the center of rotation in parallel-beam tomography. Opt. Express.

[8] Goscinski, W.J., McIntosh, P., Felzmann, U., Maksimenko, A., Hall, C.J., Gureyev, T., Thompson, D., Janke, A., Galloway, G., Killeen, N.E.B., Raniga, P., Kaluza, O., Ng, A., Poudel, G., Barnes, D.G., Nguyen, T., Bonnington, P., Egan, G.F.: The multi-modal Australian ScienceS imaging and visualization environment (MASSIVE) high performance computing infrastructure: Applications in neuroscience and neuroinformatics research. Front. Neuroinform. 8(MAR), 30 (2014) 10.3389/fninf.2014.00030 PMC3973921 24734019

[9] Brun, F., Accardo, A., Kourousias, G., Dreossi, D., Pugliese, R.: Effective implementation of ring artifacts removal filters for synchrotron radiation microtomographic images. International Symposium on Image and Signal Processing and Analysis, ISPA **672–676**, (2013)

[10] Bukreeva I, Mittone A, Bravin A, Festa G, Alessandrelli M, Coan P, Formoso V, Agostino RG, Giocondo M, Ciuchi F, Fratini M, Massimi L, Lamarra A, Andreani C, Bartolino R, Gigli G, Ranocchia G, Cedola A (2016). Virtual unrolling and deciphering of herculaneum papyri by X-ray phase-contrast tomography. Sci. Rep..

[11] Brun F, Pacilé S, Accardo A, Kourousias G, Dreossi D, Mancini L, Tromba G, Pugliese R (2015). Enhanced and flexible software tools for X-ray computed tomography at the italian synchrotron radiation facility elettra. Fundam. Informaticae.

[12] Tromba G, Longo R, Abrami A, Arfelli F, Astolfo A, Bregant P, Brun F, Casarin K, Chenda V, Dreossi D, Hola M, Kaiser J, Mancini L, Menk RH, Quai E, Quaia E, Rigon L, Rokvic T, Sodini N, Sanabor D, Schultke E, Tonutti M, Vascotto A, Zanconati F, Cova M, Castelli E (2010). The SYRMEP beamline of Elettra: clinical mammography and bio-medical applications. AIP Conference Proceedings.

[13] Gürsoy D, De Carlo F, Xiao X, Jacobsen C (2014). TomoPy: a framework for the analysis of synchrotron tomographic data. J. Synchrotron Radiat..

[14] van Aarle W, Palenstijn WJ, De Beenhouwer J, Altantzis T, Bals S, Batenburg KJ, Sijbers J (2015). The ASTRA toolbox: a platform for advanced algorithm development in electron tomography. Ultramicroscopy.

[15] Pelt DM, Gürsoy D, Palenstijn WJ, Sijbers J, De Carlo F, Batenburg KJ (2016). Integration of TomoPy and the ASTRA toolbox for advanced processing and reconstruction of tomographic synchrotron data. J. Synchrotron Radiat..

[16] Buzug TM (2008). Computed Tomography: From Photon Statistics to Modern Cone-Beam CT.

[17] Mirone A, Brun E, Gouillart E, Tafforeau P, Kieffer J (2014). The PyHST2 hybrid distributed code for high speed tomographic reconstruction with iterative reconstruction and a priori knowledge capabilities. Nucl. Instrum. Methods Phys. Res. Sect. B: Beam Interact.Mater. Atoms.

[18] Van Nieuwenhove V, De Beenhouwer J, De Carlo F, Mancini L, Marone F, Sijbers J (2015). Dynamic intensity normalization using eigen flat fields in X-ray imaging. Opt. Express.

[19] Sijbers J, Postnov A (2004). Reduction of ring artefacts in high resolution micro-CT reconstructions. Phys. Med. Biol..

[20] Raven C (1998). Numerical removal of ring artifacts in microtomography. Rev. Sci. Instrum..

[21] Boin M, Haibel A (2006). Compensation of ring artefacts in synchrotron tomographic images. Opt. Express.

[22] Münch B, Trtik P, Marone F, Stampanoni M (2009). Stripe and ring artifact removal with combined wavelet—Fourier filtering. Opt. Express.

[23] Oimoen, M.J.: An effective filter for removal of production artifacts in U.S. Geological Survey 7.5-minute digital elevation models. Proceedings of the Fourteenth International Conference on Applied Geologic Remote Sensing, Las Vegas, Nevada, 311–319 (2000)

[24] Moosmann J, Hofmann R, Baumbach T (2011). Single-distance phase retrieval at large phase shifts. Opt. Express.

[25] Irvine S, Mokso R, Modregger P, Wang Z, Marone F, Stampanoni M (2014). Simple merging technique for improving resolution in qualitative single image phase contrast tomography. Opt. Express.

[26] Weitkamp T, Haas D, Wegrzynek D, Rack A (2011). ANKAphase: software for single-distance phase retrieval from inline X-ray phase-contrast radiographs. J. Synchrotron Radiat..

[27] Hofmann R, Moosmann J, Baumbach T (2011). Criticality in single-distance phase retrieval. Opt. Express.

[28] Pacilé S, Brun F, Dullin C, Nesterets YI, Dreossi D, Mohammadi S, Tonutti M, Stacul F, Lockie D, Zanconati F, Accardo A, Tromba G, Gureyev TE (2015). Clinical application of low-dose phase contrast breast ct: methods for the optimization of the reconstruction workflow. Biomed. Opt. Express.

[29] Pelt DM, Batenburg KJ (2014). Improving filtered backprojection reconstruction by data-dependent filtering. IEEE Trans. Image Process..

[30] Pelt, D.M., Batenburg, K.J.: Accurately approximating algebraic tomographic reconstruction by filtered backprojection. In: Proceedings of The 13th International Meeting on Fully Three-Dimensional Image Reconstruction in Radiology and Nuclear Medicine, pp. 158–161 (2015)

